# Canine Alveolar Echinococcosis: An Emerging and Costly Introduced Problem in North America

**DOI:** 10.1155/2023/5224160

**Published:** 2023-02-21

**Authors:** Temitope U. Kolapo, Allison Hay, Karen M. Gesy, Caroline F. Frey, Jamie L. Rothenburger, Danny J. Joffe, Tim Spotswood, Yanyun Huang, Alessandro Massolo, Andrew S. Peregrine, Janet E. Hill, Emily J. Jenkins

**Affiliations:** ^1^Department of Veterinary Microbiology, Western College of Veterinary Medicine, University of Saskatchewan, Saskatoon, Canada; ^2^Department of Large Animal Clinical Sciences, Western College of Veterinary Medicine, University of Saskatchewan, Saskatoon, Canada; ^3^Institute of Parasitology, Department of Infectious Diseases and Pathobiology, Vetsuisse Faculty, University of Bern, Länggassstrasse 122, CH-3012 Bern, Switzerland; ^4^Faculty of Veterinary Medicine and Canadian Wildlife Health Cooperative (Alberta Region), University of Calgary, 3280 Hospital Dr. NW, Calgary AB T2N 4Z6, Canada; ^5^VCA Canada CARE Centre, Calgary, Canada; ^6^Prairie Diagnostic Services Inc., Saskatoon, SK, Canada; ^7^Ethology Unit, Department of Biology, University of Pisa, Pisa 56126, Italy; ^8^Faculty of Veterinary Medicine, University of Calgary, Calgary, Alberta T2N 1N4, Canada; ^9^UMR CNRS 6249 Chrono-Environnement, Université Bourgogne Franche-Comté, Besançon 25030, France; ^10^Department of Pathobiology, Ontario Veterinary College, University of Guelph, Guelph, Ontario, Canada

## Abstract

Alveolar echinococcosis (AE), caused by the metacestode of *Echinococcus multilocularis*, is emerging in both dogs and people in North America. Here, we review 27 cases of canine AE opportunistically reported since the index case was described in 2009 in Western Canada. We describe clinical presentation, diagnosis, treatment, outcome, and source of canine infection, based on genetics of the parasite isolated from some canine cases. Diagnosis of AE was by histopathology and/or PCR on DNA extracted from metacestodes. The median age of dogs at diagnosis was 4 years (range 1–12), which is low compared to neoplasia, the most common differential diagnosis. There was no sex predilection and different breeds were involved, but there were a disproportionate number of boxers and beagles relative to their representation in the general canine population. The most common potential risk factors included contact with wildlife and visits to off leash areas. Abdominal distension was the most common clinical sign at presentation, and medical imaging generally revealed an abdominal mass. On histopathology, protoscoleces were observed in 7 out of 14 dogs. In 7 cases, DNA sequences were most similar to European (versus North American) haplotypes, identical to those recently reported in coyotes as definitive hosts in North America, and different between eastern and western North America, implying multiple introduction events. Dogs that were not treated (*n* = 6) had 16% survival in the first 100 days in comparison with 82% survival of treated dogs (*n* = 11). Direct costs to the owner of treating canine AE ranged from 1,317 to 12,655 CAD depending on the situation at the onset of treatment. This study provides important clinical, epidemiological, and economic information for veterinary practitioners and regulators for importation of dogs, and for public health, as dogs with AE may serve as indicators of parasite range expansion and risk to humans.

## 1. Introduction

Alveolar echinococcosis (AE) is a serious extraintestinal parasitic infection caused by the metacestode stage of *Echinococcus multilocularis* [1]. Adult tapeworms develop in the intestine of canid definitive hosts where they release eggs passed with feces. Eggs are immediately infective and contaminate the environment, where they are ingested by rodent intermediate hosts [1, 2]. Humans act as aberrant intermediate hosts, developing AE after accidentally ingesting eggs [3]. While dogs usually act as definitive hosts after ingesting a metacestode in the liver of a rodent, they occasionally also develop AE following ingestion of eggs or through autoinfection [4, 5]. In dogs, AE is debilitating and associated with a poor prognosis, especially if diagnosed late due to nonspecific signs and similarity to hepatic neoplasia [6–8]. Diagnosed cases usually require treatments which can be lifelong, leading to animal welfare concerns, as well as financial and emotional burden on the dog owners.

*Echinococcus multilocularis* is a well-recognized cause of human AE in parts of Europe including Switzerland, eastern and central France, southern Germany, and western Austria, with several canine AE cases described [5, 7–10]. In North America, the north central USA and prairie and arctic regions of Canada have long been considered endemic for *E*. *multilocularis* [11, 12]; however, only recently has canine AE been recognized in North America, in regions outside the traditional distribution, like British Columbia [13, 14], Ontario [15–17], and the eastern United States [18]. This emergence has been attributed to the introduction of European-type (versus endemic North American) strains with broader host range [13, 14], and/or range expansion from existing areas of endemicity [13, 15]. Neither the USA nor Canada have any regulations in place requiring testing or treatment of imported dogs for *Echinococcus* spp. (see CFIA and CDC regulations for dog importation https://inspection.canada.ca/importing-food-plants-or animals/pets/eng/1326600389775/1326600500578; https://www.cdc.gov/importation/bringing-an-animal-into-the-united-states/apply-dog-import-permit.html), which is unfortunate, as European-type strains of *E. multilocularis* may have broader zoonotic potential and/or more pathogenicity in dogs and people [19, 20]. Interestingly, most of the AE-affected dogs had no travel history, suggesting local transmission, possibly due to ingestion of eggs in feces from wildlife such as foxes, wolves, and coyotes [11, 21, 22]. Coyotes and their feces are increasingly common not only in rural areas but also in urban green spaces and dog parks [23]. Prevalence of *E*. *multilocularis* in coyotes ranged from 25%–65% in Alberta [22, 24] to 72% in Saskatchewan [25]. Therefore, there is a pressing need to raise awareness of this parasite in North America due to rising numbers of locally acquired human cases [26, 27], high prevalence in local wildlife, spread of the parasite to new areas, the cryptic and chronic nature of the disease, poor clinical outcomes if not detected early in both dogs and people, and the preventable nature of infection of dogs with the adult tapeworms.

Here, we summarise a retrospective case series of canine AE cases in Western Canada since the initial case was described in North America (2009–2021), describing the clinical manifestations, epidemiology, treatment, and outcome of infection. We hypothesized that most cases would be from Alberta and Saskatchewan, given the high prevalence in wildlife and detection of human AE cases in these provinces. We used molecular epidemiological approaches to determine strains of the parasite causing canine AE in our study, hypothesizing that these would be identical to those from most humans and coyotes in North America (i.e., European-like strains). We also administered questionnaires to submitting veterinarians and pathologists to determine potential sources of exposure. Finally, compared to human AE where direct cost of treatment in a recent study in Austria was 30,832€–62,777€ for a 10-year period of treatment [28]; there was no published information on the cost of treating canine AE. Therefore, we did a direct health care cost analysis estimate for canine AE under four possible scenarios. Our aim was to provide data for veterinarians and dog owners to help minimize risk of exposure and to optimally manage cases of canine AE, and to support evidence-based decision making towards the establishment of control measures by policy makers to prevent further introduction of other strains and species of *Echinococcus* with significance for veterinary and public health.

## 2. Materials and Methods

### 2.1. Case Identification

We identified cases by contacting provincial diagnostic laboratories in Western Canada, including Prairie Diagnostic Services Inc. (PDS) in Saskatoon, Saskatchewan; Diagnostic Services Unit at the University of Calgary in Calgary, Alberta, Faculty of Veterinary Medicine; Animal Health Laboratory in Abbotsford, British Columbia; and Province of Manitoba Veterinary Diagnostic Services in Winnipeg, Manitoba. Veterinary pathologists were asked to provide reports and contact information for referring veterinarians from any suspect or confirmed cases of canine AE. Referring veterinarians were contacted via e-mail or telephone and asked to provide information on the case and complete a questionnaire. Additional cases were identified from veterinarians via word of mouth. Some owners provided consent to the referring veterinarian to be contacted directly. As questionnaires dealt solely with animal risk factors and our primary contacts were referring veterinarians and pathologists, we were not required to hold human or animal research ethics for this case review. All identifying information for the cases was removed. Available demographics (age, sex, breed, and province) of each dog who met the inclusion criteria were recorded and the association between sex and AE infection was determined using chi-square (MedCalc statistical software version 19.2.6 (MedCalc Software Ltd, Ostend, Belgium; https://www.medCalc.org; 2020) with a significance level set to 0.05.

### 2.2. Diagnosis

Cases were defined as possible or confirmed AE infections following the classification system outlined by Brunetti et al. [29]. Possible cases were those which presented with clinical signs suggestive of AE (distended abdomen; palpably enlarged liver; and suggestive imaging findings such as abdominal organomegaly on radiographs, multiple cavitated hepatic masses on CT and ultrasound, echogenic hepatic, and sometimes mesenteric masses with variable cavitation on ultrasound), or positive serology. Cases were confirmed by histopathology or polymerase chain reaction (PCR). Veterinary pathologists identified AE lesions histologically based on multilocular cystic structures lined with eosinophilic hyaline membranes, intraluminal protoscoleces or hooklets, and basophilic calcareous corpuscles. PCR on samples such as abdominal fluid and/or tissue from liver lesions of affected dogs was performed in research settings including the University of Calgary Faculty of Veterinary Medicine, Western College of Veterinary Medicine, University of Guelph, or by a commercial laboratory (Antech Diagnostics, Canada). When possible, we requested or performed a modified Stoll's centrifugation sucrose flotation on feces from AE dogs prior to treatment to determine if taeniid eggs (i.e., *Echinococcus* spp. or *Taenia* spp.) were present.

### 2.3. Treatment and Clinical Outcomes

Data on the treatment type (i.e., surgical resection, drug therapy, or a combination of both) were collected along with the outcome of infection and the time span from presentation to outcome (euthanized, dead, or alive). Survivability was estimated using the Kaplan–Meier analysis [30].

### 2.4. Molecular Analysis

When available, DNA was extracted from fresh or frozen abdominal fluid and/or liver lesion tissues of dogs (*n* = 16), using a commercial kit (Qiagen DNeasy Blood and Tissue Kit) and assayed using a multiplex PCR targeting a 395 bp region of the NADH dehydrogenase subunit 1 (NAD 1) mitochondrial gene of *E. multilocularis* [31]. We also tested feces of one dog with AE that had taeniid eggs in feces using this multiplex PCR. Following this screening PCR to diagnose *E. multilocularis*, we did a haplotyping PCR on 11 canine AE samples (10 from Western Canada and one previously published case from Southern Ontario) [16], using primers targeting the NADH dehydrogenase subunit 2 (NAD2) mitochondrial gene [19]. PCR products were resolved by electrophoresis using 1.5% agarose gel and purified using the QIAquick PCR Purification Kit (Qiagen Inc., Valencia, California, USA). Purified products were sequenced using the amplification primers (National Research Council Canada) and the resulting NAD2 raw sequences from 7 of the 11 samples were edited and assembled using Staden Package software (version 1.5) and aligned to published sequences in GenBank. To create a haplotype network, trimmed NAD2 sequences were aligned with reference sequences from Nakao et al. [19], a human AE case in Canada (GenBank accession number: MT250265) [26], adult cestodes in coyotes in Saskatchewan (MT250266, KC55008) [26], metacestodes in deer mice in Saskatchewan (KC549993) [32], as well as AE in a dog from the eastern United States (MT250267) [18]. Multiple sequence alignment was done via ClustalW and viewed using CLC sequence viewer 8.0 while the haplotype network was created using the statistical parsimony-based algorithm based on the NAD2 mitochondrial gene (846 bp alignment) implemented in PopART (http://popart.otago.ac.nz/index.shtml).

### 2.5. Direct Health Care Cost for Canine Alveolar Echinococcosis (AE)

Cost estimates were obtained from veterinary clinicians at a referral veterinary medical centre (as most owners willing to treat canine AE would probably accept referral), and we accessed information on the financial cost of treating canine AE under four common scenarios. We based our analysis on a 5–10 year life expectancy since most AE treatments are lifelong and the median age of diagnosis was 4 years in our case series. Broadly, we estimated costs based on direct health care costs (i.e., costs billed to the client) as listed by Jo [33], with no consideration for indirect costs.

## 3. Results

### 3.1. Dogs and Clinical Presentation

A total of 27 dogs (24 confirmed and 3 possible cases) were included in the study. The dogs presented from British Columbia (*n* = 1), Alberta (*n* = 18), Saskatchewan (*n* = 6), and Manitoba (*n* = 2). The median age at the diagnosis was 4 years (range 1–12 years). A variety of breeds were represented, the most common being the Boxer (*n* = 5), Labrador Retriever (*n* = 4), Beagle (*n* = 3), and German Shepherd (*n* = 3). We could not detect any association with sex (14 females, 13 males: *X*^2^ = 0.037, d*f* = 1, *p* = 0.847). Seven out of 14 dogs that had histology carried out had protoscoleces identified on histopathology (Table 1). The most common presenting clinical sign was abdominal distension (15 dogs) followed by vomiting (7 dogs), lethargy (6 dogs), weight loss (5 dogs), inappetence (4 dogs), diarrhea (3 dogs), pain on abdominal palpation (3 dogs), seizures (2 dogs), fever (1 dog), polyphagia (1 dog), polydipsia (1 dog), and inappropriate urination (1 dog). The most common abnormalities identified with serum chemistry were elevated globulins (10 dogs; 37.0%), increased alkaline phosphatase (ALP; 8 dogs; 29.6%), increased alanine amino transferase (ALT; 7 dogs; 25.9%), and decreased albumin (7 dogs; 25.9%). We observed associations of elevated globulin with decreased albumins in 4 cases.

### 3.2. Data on Potential Risk Factors for Canine AE

Questionnaires for 20 out of the 27 cases were completed and returned. Of the likely risk factors for exposure, foxes and coyotes were observed in the area for 15/20 dogs, 14/20 dogs visited off leash areas, 13/20 had contact with wildlife, 10/20 were coprophagic, 6/20 were on raw diets, 5/18 were known to eat rodents, 7/18 had unsupervised time off leash and it was uncertain if rodents had ever been consumed, and 6/18 were thought to have never consumed rodents. Only 3 dogs had a history of deworming with praziquantel (helps reduce risk of environmental contamination with eggs and human AE when dogs have intestinal adult worm infection). Five dogs had fecal analysis using centrifugal flotation prior to or at the time of diagnosis of AE, and only one had taeniid eggs identified in their feces, which proved to be *Taenia* spp. on multiplex PCR.

### 3.3. Clinical Presentation and Diagnosis

**Figure 1:**
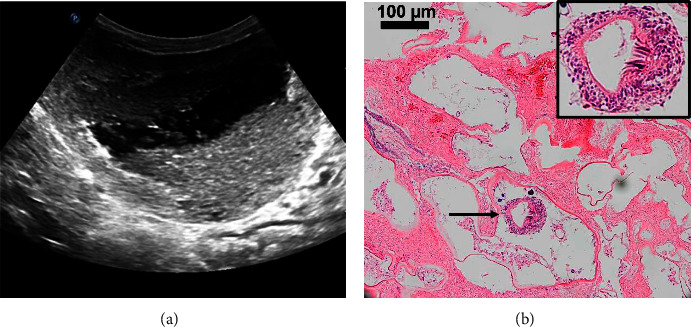
(a) Ultrasound image of the liver showing cavitated mass containing echogenic dependent and variably shadowing sediment, a classic presentation of AE; (b) histopathology of a dog liver with the presence of multilocular cysts diagnostic of AE. Thick arrow delineates a protoscolex (see inset).

Grossly, all the dogs (7 at surgery, 20 at necropsy) had severe hepatomegaly, multifocal to coalescing liver lesions/cysts ranging from 5–150 mm in diameter. These cysts contained abundant yellow-white friable materials with smaller amounts of flocculent grey fluid. Aspirates of the cyst material contained protoscoleces (7/14) consistent with a metacestode. On ultrasound, the most common findings included multiple cavitated masses, hyperechoic thickened capsules, and hypoechoic heterogenous fluid-filled cavities (up to >6 litres in one case) (Figure 1(a)). However, sonographic appearance of AE was variable, with some cases presenting with solid lesions devoid of cavitation, with variably echogenic multinodular (<2 cm) masses with shadowing, mineralization, and/or acoustic enhancement (“hail-storm” pattern described elsewhere) [6].

DNA of *E. multilocularis* was detected in fresh or frozen samples (abdominocentesis fluid, cyst fluid, and/or liver tissue from surgical biopsies or necropsies) from 16 dogs for which samples were available. Hepatic histopathology was described in 14 dogs, where intralesional calcareous corpuscles compatible with a cyclophyllid metacestode were present with multiple cysts separated by bands of fibrosis and granulomatous inflammation. The cysts were lined by thick hyaline capsules and often filled with proteinaceous fluid, degenerative cellular debris, neutrophils, and calcareous corpuscles. Rarely, fragments or entire protoscoleces were identified, sometimes enclosed within small granulomas within the fibrous tissue around the larger coalescing cysts (Figure 1(b)). Most cases were diagnosed based on compatible gross lesions confirmed with PCR (Table 1).

### 3.4. Extent of AE Lesions

Liver involvement was well described in 21 cases, including the right side (*n* = 4), left side (*n* = 5), and both right and left aspects of the liver (*n* = 12). In six cases, descriptions of liver involvement were not possible due to incomplete imaging data. Macroscopic metastasis to other abdominal organs was evident in 10 cases seen at surgery and necropsy and involved the hepatic lymph nodes, peritoneum, mesentery, pancreas, diaphragm, stomach, and spleen. Distant metastasis was observed in the lungs and lymph nodes (location not specified) of four cases using imaging techniques and at necropsy (Table 2). Pyothorax was found in one dog; however, association with AE was unclear.

### 3.5. Treatment and Outcome

**Figure 2:**
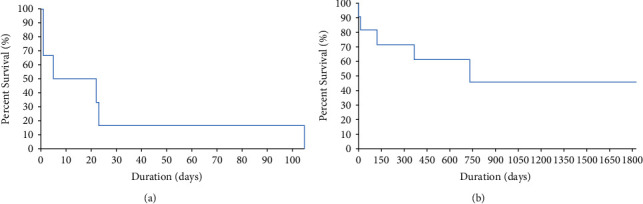
Kaplan–Meier survival curves for dogs with alveolar echinococcosis that were (a) untreated, and (b) treated (medical, surgical, or both) in 17 dogs in Western Canada, 2009–2021.

Complete data on treatment and outcome (euthanized, dead, or alive) were available for 17 dogs (6 untreated and 11 treated) of which 15 were confirmed and 2 were possible, and these were included in the analysis. For dogs on medication, the most common regimen was 10 mg/kg of albendazole daily (Table 2). Dogs that were not treated had 50% survival in the interval of 5–22 days, and this reduced to 16% survival in the interval of 23–108 days. In contrast, treated dogs had 82% survival in the interval of 20–119 days, which reduced to 46% survival in the interval of 740–1825 days (Figure 2).

### 3.6. Haplotype Analysis

**Figure 3:**
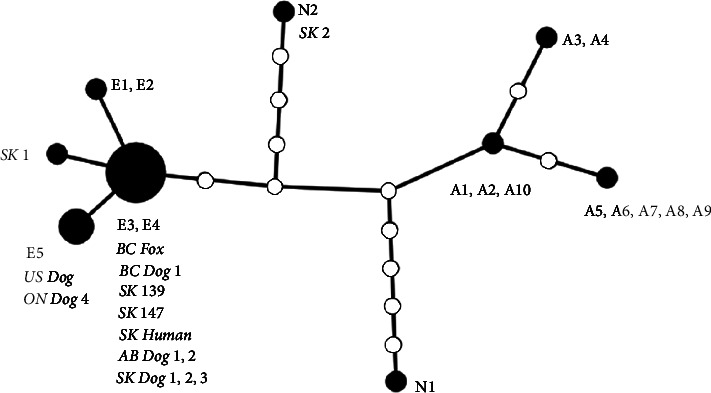
Statistical parsimony-based haplotype network based on the NAD2 mitochondrial gene (846 bp alignment) of *Echinococcus multilocularis*. Haplotypes in bold (A1-A10, N1, N2, and E1-E5) are from elsewhere [19]. Haplotypes in italics are previously described in coyotes from Saskatchewan (SK 1, SK 139, and SK 147), fox from British Columbia (BC fox), Saskatchewan rodents (SK 2), a dog from Virginia USA (US dog), and a human AE case from Saskatchewan (SK human). Italicised bold isolates are from dogs in the current study (BC dog 1; AB dog 1 and 2; SK dog 1, 2, and 3) and a previously published case in a dog from Ontario (ON dog 4). Unlabelled white circles represent a single nucleotide change from adjacent sequences.

DNA of *E. multilocularis* from 7 dogs, including the index case in Canada from British Colombia, two Alberta dogs and three Saskatchewan dogs in the current study, and one Ontario dog (previously published case), were successfully sequenced at the NAD2 locus. All the dog isolates except one grouped with the E3/E4 haplotype (originally reported from a red fox in France, accession number AB461404), similar to coyotes from SK (KC55008 and MT250266), a fox from BC, and a human case from SK (MT250265). One dog isolate (ON Dog 4) from Ontario grouped with the E5 haplotype (originally reported in red fox from Slovakia, accession number AB461414), similar to a dog from Virginia in the USA (accession number MT250267) (Figure 3).

### 3.7. Direct Health Care Cost for Canine Alveolar Echinococcosis

The cost of treating canine AE can vary greatly depending on the prevailing situation at the time of diagnosis.  Table 3 summarises the estimated cost of treatment under 4 possible scenarios while full details can be found in Table S1.

## 4. Discussion

We summarised clinical features, diagnosis, risk factors, molecular epidemiology, and management of the first described cases of canine AE in Western Canada, from 2009 to 2021. The typical case was a young adult dog, possibly a boxer, retriever, or beagle, with a history of off leash walks in a region where red fox and/or coyote are frequently observed. Most cases presented with abdominal distension and medical imaging revealed an abdominal mass, and the most common differential diagnosis was neoplasia. On resection, histopathology revealed typical protoscoleces in only half of cases, with PCR being the preferred method of the definitive diagnosis. On further genetic characterization, the parasite was identical to that present in local wild canids and were European, versus North American, haplotypes. Treatment increased survival in the first 100 days dramatically (from 16% to 82%) but cost to the owner, on average, would be about $10,000 Canadian dollars. Note that this is an underestimate, as it does not include indirect costs due to loss of time of pet owners, loss in canine quality of life, or pain and suffering of the pet owner associated with lifelong management and poor outcomes of canine AE. We also did not estimate costs to public health, as every canine AE case indicates a certain amount of environmental contamination and therefore risk of human exposure.

As canine AE is emerging in North America, awareness among veterinarians and pet owners is critical to encourage prompt diagnosis and swift action for better clinical outcomes. We provide further evidence that European strains of the parasite, which may be more zoonotic and/or virulent than the North American strains, are spreading among dogs, wildlife, and people. Finally, we identified potential risk factors for canine AE to enable better client and public education, as well as considerations for creating useful control programs.

The majority of cases in this study were from the province of Alberta, followed by Saskatchewan. This finding was not entirely surprising, as both provinces have, for the first time in 2013 in Alberta, recorded locally acquired cases of AE in humans [26, 27, 34] as well as a high prevalence of *E. multilocularis* in coyotes [22, 25, 35]. Furthermore, coyotes are increasingly found within urban communities close to homes and in dog parks [36]. Most of the affected dogs in this study had contact with wildlife (specifically coyotes and/or foxes), visited off leash areas, and were coprophagic, all potential risk factors for canine AE. The high prevalence of the parasite in coyotes [23, 26], low prevalence in dogs as definitive hosts in Canada [37], and our observation that none of the dogs were infected with adult cestodes at necropsy or shedding eggs of *E. multilocularis* in feces at the time of AE diagnosis suggests that dogs were likely infected directly from environments contaminated with feces of other canids. Taeniid eggs found in feces of one dog with AE were identified as *Taenia* spp. by PCR, further emphasizing the usefulness of coproPCR for sensitive and specific detection and distinction of *E. multilocularis* from other related tapeworm species in dogs as definitive hosts.

We report several breeds of dogs affected by AE, which may largely reflect the most common breeds in the region; however, boxers, which are not among the most common breeds kept as pets in Canada (https://www.readersdigest.ca/home-garden/pets/most-popular-dog-breeds-in-canada/; https://wagwalking.com/breed/top-dog-breeds-in-canada), ranked the highest, along with Golden Retrievers, Beagles, and German Shepherds. These breeds might be more susceptible because of their genetics [5], or their natural behaviour of following scent trails (beagles) and picking up things in their mouth (retrievers), which predispose them to ingest infective eggs in the environment. Also, large breeds of dogs may be more likely to be allowed off leash and spend more time outside compared to small or toy breeds. Consistent with a previous study in dogs in Europe, there were no statistically significant difference between male and female dogs in our case series [8].

AE presents with nonspecific clinical signs, and gross lesions could be initially confused with neoplasia and/or abscesses [17], as well as other space-occupying lesions in the liver. Clinicians may not initially consider AE as a differential diagnosis, especially in areas where the disease is newly emerging, which can lead to poor clinical outcomes as seen in this case review as well as in Europe [8]. Therefore, suggestive diagnostic imaging should be followed up with further testing to confirm diagnosis and to stage the clinical progression of the disease. CT is an increasingly important modality for evaluating space-occupying hepatic and other abdominal lesions, especially for planning surgery. CT is also the modality of choice for detecting pulmonary lesions, which were detected in 3 dogs in this series, including nodular pulmonary infiltrates which had not been previously described in the literature.

Genetic haplotypes among *E. multilocularis* have been linked to the geographic origin [19], and the North American N2 strain native to prairie regions of Canada and the north central USA was not detected in any AE cases. The presence of European-type strains in humans, wild canids [26], and now dogs with AE that have not travelled outside Western Canada provide strong evidence for the establishment of these “European-type” strains in North America. In the present study, the dominant haplotype in Western Canada (E3/E4) was distinct from the E5 haplotype identified in dogs in eastern North America (Ontario, Canada [16], and Virginia, USA [18]). This suggests that multiple introduction events in North America are likely and indeed not surprising in light of the absence of any mandatory testing or treatment for parasites on import of dogs in North America. Considering the increasing reports of locally acquired human AE in Canada [11, 26, 27, 34], detection of canine AE in newly endemic regions [14, 15, 18], as well as high prevalence (30–70%) in wild canid definitive hosts [22, 25, 35], AE appears to be emerging in dogs and people in North America and regulatory efforts should be directed toward prevention of importation of other strains and species of *Echinococcus*, including highly pathogenic Asian strains of *E. multilocularis* [19, 38] and pastoral strains of the *E. granulosus* species complex.

Better access to serological and medical imaging screening of dogs for AE is needed in North America, especially for dogs at higher risk of infection. This will more accurately estimate the true prevalence of canine AE in North America, as the number of cases presented here is likely a significant underestimate. Our method of recruiting cases may have favoured certain dog populations. For instance, not all owners of dogs affected by AE may have sought veterinary care or pursued diagnostic tests, which can be prohibitively expensive. In addition, some affected dogs may have died or been euthanized without further investigations due to welfare considerations. Even if necropsies are performed in clinics, misdiagnosis as neoplasia can occur sometimes, and clinicians may not be authorized by clients to perform molecular tests. In addition, surgical biopsy specimens are most often fixed in formalin, reducing their utility for PCR and thus the number of isolates available for molecular diagnostics. These tests are only starting to become commercially available in North America, further contributing to underdiagnosis. Finally, underreporting remains a significant issue, as *E. multilocularis* in animals is not nationally reportable to animal or public health authorities in Canada or the USA, although laboratory-diagnosed cases are annually notifiable to the World Organization for Animal Health (see CFIA, CDC, and USDA on nationally reportable diseases).

Albendazole is the drug of choice for the treatment of AE [39], but two dogs in this case study were treated with fenbendazole, another benzimidazole. While fenbendazole like albendazole is not specifically labelled as treatment for AE, it has been used experimentally in mice models with comparable efficacy, and thus can be considered a potential alternative treatment for AE [40]. Most of the dogs in this study were not dewormed regularly against tapeworms. Praziquantel, the only drug labelled against *Echinococcus* spp. in North America, is an adult cestocide with no prophylactic action against developing canine AE [41, 42]. However, routine monthly deworming with praziquantel in high-risk regions, for dogs with access to wild rodents, would likely reduce the risk of development and establishment of intestinal infections with adult *E. multilocularis*, thereby reducing contamination of the environment with infective eggs [5, 43]. This also has public health implications because humans can become infected via accidental ingestion of eggs passed in dog feces. Thus, monthly administration of praziquantel is recommended for dogs that are at a high-risk of intestinal infection with *E. multilocularis* through consumption of rodent intermediate hosts [44]. Also, strategic surveillance using sensitive coproPCR methods on high-risk dogs (both urban and rural, especially free roaming) for the prevalence of *E. multilocularis* intestinal infection should be considered, given associations between dog ownership and human AE [45]. While AE in dogs is not directly zoonotic, these infections in dogs may serve as the indication of high levels of environmental contamination and risk to humans.

## 5. Conclusion

This decade-long, retrospective analysis of AE in domestic dogs in Western Canada suggests substantial environmental contamination with infective eggs from definitive hosts, such as coyotes. These findings are also important with respect to human health risk if prompt preventive measures and control strategies are not advocated and implemented. This study calls for regulation of imported dogs to prevent introduction of pathogenic, zoonotic parasites like *Echinococcus* spp., as well as for coordinated reporting for animal and human cases of AE to monitor emergence in new areas and increases in incidence. For example, AE in both animals and people became provincially notifiable in Ontario, Canada, in 2018 following detection of cases of canine AE. Finally, further education and awareness of the public, veterinarians, and pathologists, especially in areas adjacent to endemic regions for *E. multilocularis*, is needed to prevent unnecessary financial and emotional burden for dog owners, to ensure timely diagnosis and better clinical outcomes for animal health, and for animals to serve effectively as sentinels of increased risks for public health.

## Figures and Tables

**Table 1:** Available demographics of 27 dogs diagnosed with confirmed (*n* = 24) and possible (*n* = 3) alveolar echinococcosis in Western Canada from 2009–2021.

Dog ID	Age at diagnosis (yrs)	Sex^2^	Breed	Province	Diagnosis	Diagnostic method	Protoscoleces present
1	6	MN	Maltese mix	AB	Confirmed	Histology	No
2	1.5	FS	Shepherd Cross	AB	Confirmed	Cytology and PCR	N/A
3	3.5	FS	Beagle	AB	Confirmed	Histology	Yes
4	UNK^1^	M	UNK	AB	Confirmed	Histology	No
5	4	FS	Labrador Retriever	AB	Confirmed	Histology and PCR	Yes
6	4	FS	Beagle	SK	Confirmed	PCR	N/A
7	5	M	German Shepherd	SK	Confirmed	Histology and PCR	Yes
8	5	FS	Boxer	SK	Confirmed	PCR	N/A
9	3	MN	German Shepherd	AB^3^	Confirmed	Histology and PCR	No
10	3.5	MN	Shih Tzu/Bichon Frise	BC	Confirmed	Histology and PCR	Yes
11	1	FS	Labrador Retriever	MB	Confirmed	PCR	N/A
12	2	FS	Boxer	SK	Confirmed	PCR	N/A
13	8	MN	Boston Terrier	AB	Confirmed	U/s and histology	No
14	4	M	Golden Retriever	AB^3^	Confirmed	Histology	No
15	3	F	Labrador Retriever	SK	Confirmed	PCR	N/A
16	3	MN	Boxer	AB	Confirmed	Histology	No
17	1	FS	Mixed	AB	Confirmed	Cytology and PCR	N/A
18	7	MN	Havanese	AB^3^	Confirmed	U/s, histology and PCR	Yes
19	2	FS	Beagle	AB^3^	Possible	Serology	N/A
20	3	FS	American Eskimo	AB	Confirmed	PCR	N/A
21	3	FS	Collie Mix	AB	Confirmed	U/s, histology and PCR	Yes
22	3	MN	Labrador Retriever	AB	Confirmed	U/s, serology and PCR	N/A
23	5	MN	Boxer	AB	Possible	Serology	N/A
24	3	FS	Boxer	AB	Confirmed	Histology	Yes
25	12	MN	Akita	AB	Possible	Radiograph and U/s	N/A
26	3	FS	Mixed	MB	Confirmed	PCR	N/A
27	3	MN	Golden Retriever	SK	Confirmed	Histology and PCR	No

^1^UNK = unknown; ^2^M = male; MN = male neutered; F = female; FS = female spayed; ^3^Travel outside of Canada took place prior to diagnosis; U/s = ultrasound; PCR = polymerase chain reaction.

**Table 2:** Diagnosis, therapy, and time from presentation to outcome in 27 dogs with confirmed (*n* = 24) and possible (*n* = 3) alveolar echinococcosis in Western Canada, 2009–2021.

Case #	Lesions (size)	Free abdominal fluid	Abdominal metastasis	Distant metastasis	Surgical intervention	Medical therapy	Time to outcome (days)	Outcome
1	Multiple	Yes (<50 mL)	Peritoneum	None	None	Fenbendazole (10 mg/kg daily)	Unknown	Unknown
2	Multiple (4–10 cm)	Yes (40 mL)	Hepatic lymph node	Lungs	None	None	23	Euthanasia
3	Multiple (2 mm–7 cm)	Yes (1.5 litres)	None	None	None	None	22	Euthanasia
4	Multiple (up to 15 cm)	Yes (10 mL)	Omentum	Lymph nodes	None	None	1	Euthanasia
5	Multiple	Yes (3.5 litres)	Hepatic lymph node, mesentery, and pancreas	None	Yes (partial resection)	Albendazole (10 mg/kg daily)	13	Acute death 9 days after discharge from surgery
6	Multiple	Yes (amount NA)	None	Unknown	None	Albendazole (10 mg/kg daily)	Unknown	Alive
7	NA	Yes (amount NA)	None	Unknown	Exploratory surgery	None	1	Death one day postsurgery
8	Single	NA	Diaphragm and omentum	Unknown	Yes (lobectomy)	Albendazole (10 mg/kg daily)	Unknown	Alive
9	Single	Yes (>6 L)	Gall bladder	Unknown	Yes (lobectomy)	Albendazole (10 mg/kg daily)	1825	Alive
10	Single	No	Stomach	None	Yes (partial resection)	Albendazole (10 mg/kg daily)	Unknown	Survived for years
11	Single	Unknown	Unknown	Unknown	Unknown	Unknown	Unknown	Unknown
12	Single (10 cm)	No	None	None	None	Albendazole (50 mg/kg daily)	365	Euthanasia
13	Multiple	Unknown	Unknown	Unknown	None	None	1	Euthanasia
14	Multiple	Yes (amount NA)	Unknown	Unknown	None	None	Unknown	Euthanasia
15	Multiple	Yes (20 mL)	Unknown	Unknown	None	None	Unknown	Died acutely
16	Single	Yes (moderate amount)	None	None	Yes (lobectomy)	Albendazole (10 mg/kg daily)	1095	Alive
17	Multiple	Yes	Stomach	Unknown	None	Albendazole (10 mg/kg daily)	548	Alive
18	Multiple	Yes	None	None	Yes (lobectomy)	None	356	Alive
19	Single	No	Unknown	Unknown	None	Albendazole (10 mg/kg daily)	1095	Alive
20	Multiple (4 cm)	No	None	Unknown	Yes (lobectomy)	Albendazole (10 mg/kg daily)	60	Alive
21	Multiple	Yes	None	None	None	None	5	Euthanasia
22	Multiple	No	Peritoneum	Lungs	None	Fenbendazole (10 mg/kg daily)	120	Euthanasia
23	Multiple	Yes	None	None	None	Albendazole (10 mg/kg daily)	730	Euthanasia
24	Single	Yes	Unknown	Unknown	Yes	None	Unknown	Unknown
25	Single	Unknown	Unknown	Unknown	Unknown	Unknown	Unknown	Unknown
26	Single	Unknown	Unknown	Unknown	Unknown	Unknown	Unknown	Unknown
27	Multiple	Yes	Omentum, stomach	Lungs	None	None	105	Euthanasia

NA = not available.

**Table 3:** Estimated direct cost of canine AE treatment per patient based on different scenarios.

	Description	Direct costs in Canadian dollars
Scenario 1	Basic work-up, diagnosis, and euthanasia	1,317–1,590
Scenario 2	Basic work-up, diagnosis, and palliative treatment with albendazole lifelong	4,744–7,954
Scenario 3	Full work-up, diagnosis, surgical planning, surgery, hospitalization, lifelong albendazole, and no complications	8,132–11,895
Scenario 4	Full work-up, diagnosis, surgical planning, surgery, hospitalization, postop hemorrhage requiring transfusion, higher level of ICU care, and lifelong albendazole	8,846–12,655

## Data Availability

All data, not in the main manuscript, have been uploaded as supplementary information.

## Supplementary Materials

Supplementary Materials Full breakdown of the financial cost of treating canine AE under four possible scenarios (Table S1).
