# Supplementary material for: Canine Alveolar Echinococcosis: An Emerging and Costly Introduced Problem in North America
Source: Transbound Emerg Dis. 2023 Feb 21;2023:5224160. doi: 10.1155/2023/5224160 (PMC12017107; doi:10.1155/2023/5224160)
Supplement: Supplementary Materials — Full breakdown of the financial cost of treating canine AE under four possible scenarios (Table S1). [file 5224160.f1.pdf]

SUPPLEMENTARY INFORMATION

Table S1. Direct cost of canine AE treatment (Breakdown of costs)

**Scenario 1: Basic work-up, diagnosis, euthanasia**		
	**Procedure**	**Cost (Canadian dollars)**
**1**	Consultation	84-236
**2**	CBC/Chem/UA	180
**3**	IV Catheterization (IVC)	53
**4**	Sedation and Monitoring	63
**5**	Abdominal Ultrasound	365-486
**6**	Thoracic radiographs	166
**7**	U/S guided fine needle aspirate	100
**8**	Cytology	104
**9**	PCR plus shipping	133
**10**	Euthanasia	68
**11**	**Total**	**1,317-1,590**
**Scenario 2: Basic work-up, diagnosis, palliative treatment with albendazole life long**		
**1**	Consultation	84-236
**2**	CBC/Chem/UA	180.64
**3**	IV Catheterization (IVC)	53
**4**	Sedation and Monitoring	63
**5**	Abdominal Ultrasound	365-486
**6**	Thoracic radiographs	166
**7**	U/S guided fine needle aspirate	100
**8**	Cytology	104
**9**	PCR plus shipping	133
**10**	Praziquantel	17-46
**11**	***Initial Total***	***1,161-1463***
	Long term	
**12**	First year rechecks and Blood work	678.49
**13**	Subsequent years (5-10)	2097-4719
**14**	Annual imaging	647-768 (including IVC, sedation and monitoring
**15**	Albendazole/year	159-324
**16**	***Long term total***	***3,582-6,490***
**17**	**Overall total**	**4,744-7,954**
**Scenario 3: Full work-up, diagnosis, surgical planning, surgery, hospitalization, lifelong albendazole, no complication**		
**1**	Consultation	84-236
**2**	CBC/Chem/UA	180
**3**	PT	50
**4**	PIT	50
**5**	Canine blood grouping	73

**6**	IV Catheterization	53
**7**	Sedation and Monitoring	63
**8**	CT (2 sites +contrast)	1,004-1,124
**9**	U/S guided FNA	100
**10**	Cytology	104
**11**	Surgical set up (basic)	92
**12**	Anesthesia induction	326
**13**	Every following 15 mins	42*6=252
**14**	Surgical removal of lesions	500-1000
**15**	ICU admission	79
**16**	48 hours in ICU Tier 2	630
**17**	48 hours barrier nursing	158
**18**	Perioperative meds	400-500
**19**	Histopathology	199
**20**	PCR plus shipping	133
**21**	Praziquantel	17-46
**22**	***Initial total***	***4,550-5,404***
	Long term	
**23**	First year rechecks and Blood work	678
**24**	Subsequent years (5-10)	2097-4719
**25**	Annual imaging	647-768 (including IVC and sedation and monitoring
**26**	Albendazole/year	159-324
**27**	***Long term total***	***3,582-6,490***
**28**	**Overall total**	**4,744-7,954**

Scenario 4: Full work-up, diagnosis, surgical planning, surgery, hospitalization, post-op hemorrhage requiring transfusion, higher level of ICU care, life-long albendazole

**1**	Consultation	84-236
**2**	CBC/Chem/UA	180.64
**3**	PT	50
**4**	PIT	50
**5**	Canine blood grouping	73
**6**	IV Catheterization	53
**7**	Sedation and Monitoring	63
**8**	CT (2 sites +contrast)	1004-1124
**9**	U/S guided FNA	100
**10**	Cytology	104
**11**	Surgical set up (basic)	92
**12**	Anaesthesia induction	326
**13**	Every following 15 mins	42*6=252
**14**	Surgical removal of lesions	500-1000
**15**	ICU Admission	79
**16**	24 hours in ICU Tier 3	446
**17**	48 hours in ICU Tier 2	630
**18**	72 hours barrier nursing	237
**19**	1-unit PRBCs	105

**20**	Perioperative meds	400-500
**21**	Histopathology	199
**22**	PCR plus shipping	133
**23**	Praziquantel	17-46
**24**	Initial Total	5,264-6,164
	Long term	
**23**	First year rechecks and Blood work	678
**24**	Subsequent years (5-10)	2,097-4,719
**25**	Annual imaging	647-768 (including IVC and sedation and monitoring
**26**	Albendazole/year	159-324
**27**	***Long term total***	***3,582-6,490***
**28**	**Overall total**	**8,846-12,655**

Abbreviations:

CBC: complete blood count

Chem: Blood chemistry test

UA: Urinalysis

IVC: Intravenous catheterization

U/S: Ultrasound

PCR: Polymerase chain reaction

PT: Prothrombin time

PIT: Platelet count

ICU: Intensive care unit

CT: Computed tomograpy

PRBCs: Packet red blood cells
